# HANDdata – first-person dataset including proximity and kinematics measurements from reach-to-grasp actions

**DOI:** 10.1038/s41597-023-02313-w

**Published:** 2023-06-24

**Authors:** Enzo Mastinu, Anna Coletti, Samir Hussein Ali Mohammad, Jasper van den Berg, Christian Cipriani

**Affiliations:** 1 BioRobotics Institute, Scuola Superiore Sant’Anna, Pisa, Italy; 2 Delft University of Technology, Delft, Netherlands

**Keywords:** Biomedical engineering, Mechanical engineering, Translational research, Quality of life

## Abstract

HANDdata is a dataset designed to provide hand kinematics and proximity vision data during reach to grasp actions of non-virtual objects, specifically tailored for autonomous grasping of a robotic hand, and with particular attention to the reaching phase. Thus, we sought to capture target object characteristics from radar and time-of-flight proximity sensors, as well as details of the reach-to-grasp action by looking at wrist and fingers kinematics, and at hand-object interaction main events. We structured the data collection as a sequence of static and grasping tasks, organized by increasing levels of complexity. HANDdata is a first-person, reach-to-grasp dataset that includes almost 6000 human-object interactions from 29 healthy adults, with 10 standardized objects of 5 different shapes and 2 kinds of materials. We believe that such data collection can be of value for researchers interested in autonomous grasping robots for healthcare and industrial applications, as well as for those interested in radar-based computer vision and in basic aspects of sensorimotor control and manipulation.

## Background & Summary

The human hand is an incredibly complex system with a huge spectrum of functionalities, it is essential in our life and in our daily interactions with surrounding objects and people. Researchers have, for a long time, spent efforts to understand and replicate such complexity. This challenge touches upon different branches and fields of the human knowledge, for instance anatomy and biology, neuroscience, but also art and engineering, for robotics, automation, prosthetic limbs, and so forth. Such attention is demonstrated by the vast number of articles available in literature on this topic (more than half a million documents hits for a “human hand” search on Scopus!). Moreover, it is also demonstrated by the vast number of related data collections (or datasets) freely released by researchers over the years. Each of these datasets provides data from various sensors trying to focus the attention on a particular aspect related to the human hand, most often on a particular set of actions, ultimately trying to underline a different perspective of such complex system. For instance, a large number of datasets focused on the recognition of hand poses and grasps by offering various data such as RGB and depth cameras, electromyographic sensors and motion tracking systems^1–7^, even for unconstrained objects manipulations^8^. Similarly for robotics engineering, a large number of datasets focused on robot grasping automation, robot-learning, human-robot interactions, and even on ways to synthetize a grasping dataset^9–12^.

Unfortunately, only few datasets were specifically focused on the reach to grasp action^13–16^, one of the most important and repeated actions in daily life, and even fewer datasets focused on automatic grasping during reaching via computer vision^6^. Sadly, none of the currently available datasets provides extensive hand kinematics and proximity vision data during reach to grasp actions of real (i.e., non-virtual) objects.

With this work, we intended to deliver a dataset specifically tailored for studying autonomous grasping of a robotic hand, with particular attention to the reaching phase of the grasping. In details, we aimed for a data collection that would allow exploring novel autonomous grasping approaches based on arm, hand and fingers reaching trajectories estimation, as well as object characteristics recognition before and after starting the reach to grasp movement (i.e., static and dynamic phases, respectively). For such exploration, we selected a set of radar and time-of-flight proximity sensors, sensors which are more and more often seen in computer vision based technologies^17^, and with large potential for an actual clinical application in both terms of power and computational needs. Additionally, basic forearm and hand kinematics was deemed essential in order to provide a reference ground-truth for any autonomous grasping routine.

We present here and openly share with the community HANDdata^18^, to the best of our knowledge the only dataset that provides:

- a first-person focus on the reach-to-grasp action of a set of real and standardized objects,
- data comprehensive of both static (i.e., pre-reaching) and grasping scenarios organized by increasing levels of complexity,
- target object characteristics data, measured via radar and time-of-flight proximity sensors located on participants’ wrist,
- wrist and forearm kinematics data, measured via inertial sensors located on participants’ wrist,
- fingers and hand kinematics data, measured via stretch sensors located on participants’ hand,
- hand-object interaction event-related data (i.e., touch, lift-off, replace and release), measured via load cells located underneath the manipulation areas.

As the result, the HANDdata dataset includes almost 6000 human-object interactions from 29 healthy adults, with 10 standardized objects of 5 different shapes and 2 kinds of materials. This vast amount of data focus the attention on the common action of reaching and grasping an object. We sought to capture target object characteristics from radar and time-of-flight proximity sensors, as well as details of the reach-to-grasp action by looking at wrist and fingers kinematics, and at hand-object interaction main events. We structured the data collection as a sequence of tasks, moving from the most ideal static scenarios up to more dynamic ones. These tasks were performed with a collection of different objects and materials, standardized within a functional test largely known in the field of prosthetics, and meant to provide data for a limited but highly functionally relevant set of grip patterns. We believe that such data collection can support the development of models and mappings for autonomous grasping robots interacting within unconstrained environments. For instance, HANDdata can be of value for researchers exploring first-person autonomous grasping for healthcare and personal care, such as assistive and/or rehabilitation robots (e.g., prosthetic hands and surgical robots^19–21^), as well as social and industrial robots (e.g., mobile servants and warehouse collaborative robots^22–24^). Moreover, the data presented here can be of value for researchers interested in radar-based computer vision approaches for recognition of objects, and also for those interested in basic aspects of sensorimotor control and manipulation.

## Methods

### Equipment

#### Instrumented glove

**Fig. 1:**
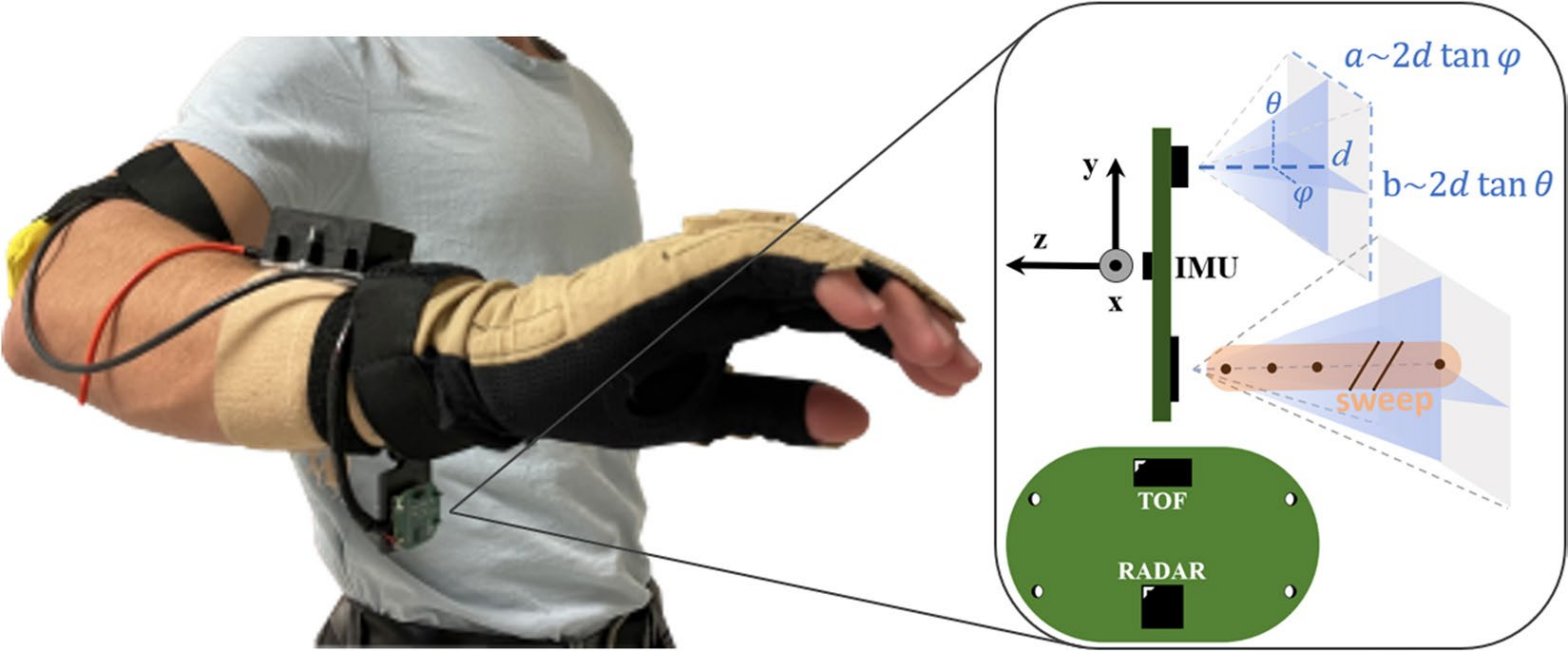
Instrumented glove. The instrumented glove used for the data collection experiment was based on a CyberGlove to which proximity and inertial sensors were added via a custom circuit board mounted on a customizable wrist band (left). Side and front view of the 40x25mm custom circuit board showing inertial sensing axes orientation as well as proximity sensors position, orientation, field-of-view triangular approximation and illustrating the concept of radar equally distributed range points constituting a sweep (right).

Volunteer participants were asked to wear an instrumented glove for the duration of the data collection experiment. The instrumented glove was based on a CyberGlove II (CyberGlove Systems, USA) to which proximity and inertial sensors were added via a 40x25mm custom circuit board mounted on a customizable wrist band (Fig. 1).

The CyberGlove was right-sided and included 18 strain gauges and data acquisition electronics which allowed tracking fingers’ joints angles with a resolution of less than one degree and a frequency of 90 Hz. Proximity sensors included a pulsed coherent radar (A121, Acconeer, Sweden) and time-of-flight sensor (VL53L5CX, STmicroelectronics, France-Italy). The A121 radar was set to emit trains of 60 GHz pulses with known starting phase in pulsing/silent cycles alternating at 13 MHz. Knowing the velocity of the emitted pulses, the radar then tried to reconstruct reflections at certain discrete distances by analysing the time taken to receive the echoes. In the configuration used in this study, the radar received pulses at times related to the distances of 35 range points, equally distributed from 6 cm to 41 cm (i.e., each range point is separated by 1 cm). Then, a complete reading of all range points, also defined as a sweep, was repeated 40 times with a rate of 800 Hz. Lastly, these 40 sweeps were collected in frames and acquired with a rate of 15 frames per second. The radar field of view covered a 3D region of approximately 65 × 53 degrees at 50% of beam power. The VL53L5CX time-of-flight sensor tried to estimate target’s distance by illuminating the region of interest with 940 nm photons (i.e., invisible light). It was set for 15 frames per second in 8 × 8 resolution (i.e., 64 pixels). Its field of view covered a 3D region of approximately 45 × 45 degrees at 75% of beam power. The inertial sensor included built-in accelerometer and gyroscope of the BMI160 (Bosch, Germany) and was set for 120 samples per second.

All data was acquired via wired USB connection through an ESP32 host MCU (EspressIf, China) directly connected to the sensors’ custom circuit board. The custom board was then attached to wrist band composed by an elastic bandage, Velcro tape and a 3D printed hinge which allowed customization of the circuit board orientation (i.e., proximity sensors field of view). The hinge angle was kept at 90° with respect to the forearm axis throughout the whole experiment.

#### Test objects and instrumented platforms

**Fig. 2:**
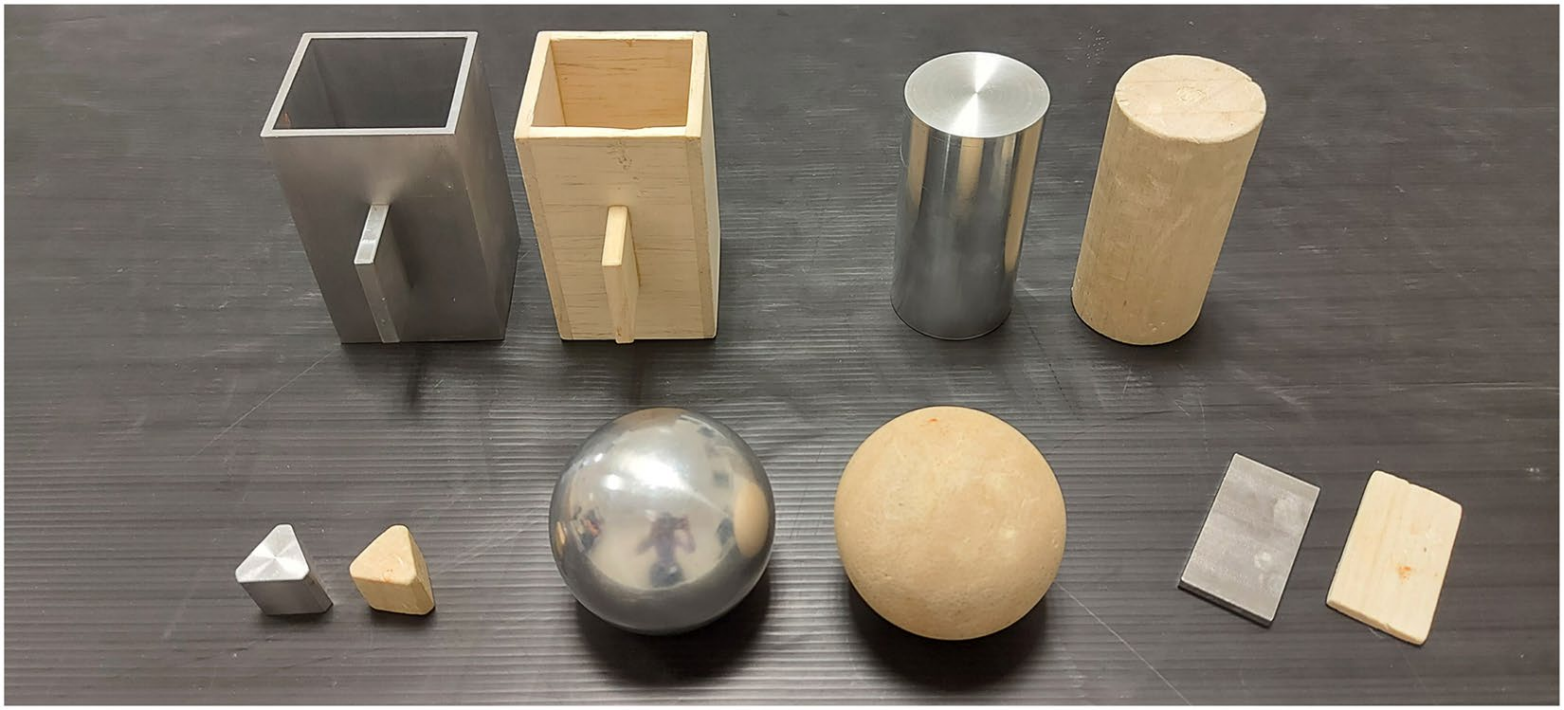
Test objects. Abstract objects like a sphere, a cylinder, a triangular prism, a cuboid with a thin rectangular prism ‘handle’ and a thin rectangular prism were meant to trigger the spherical, power, tri-digit, lateral and pinch grip pattern, respectively.

Ten different objects were used for this experiment (Fig. 2). Different shapes and materials were chosen so to allow exploring shape recognition across different permittivity constants. The test objects were selected to trigger a limited but highly functionally relevant set of grip patterns. For this, the objects were directly taken from the Southampton Hand Assessment Procedure^25^ (SHAP), a clinically validated functional assessment, well-established in the field of prosthetics. Similarly to the SHAP protocol, here each object was meant to trigger and assess a certain grip pattern. Specifically, abstract objects like a sphere, a cylinder, a triangular prism, a cuboid with a thin rectangular prism ‘handle’ and a thin rectangular prism were meant to trigger the spherical, power, tri-digit, lateral and pinch grip pattern, respectively. Each object was available in two materials, wood or aluminium. Objects dimensions, materials and weights are standardized within the SHAP protocol.

**Fig. 3:**
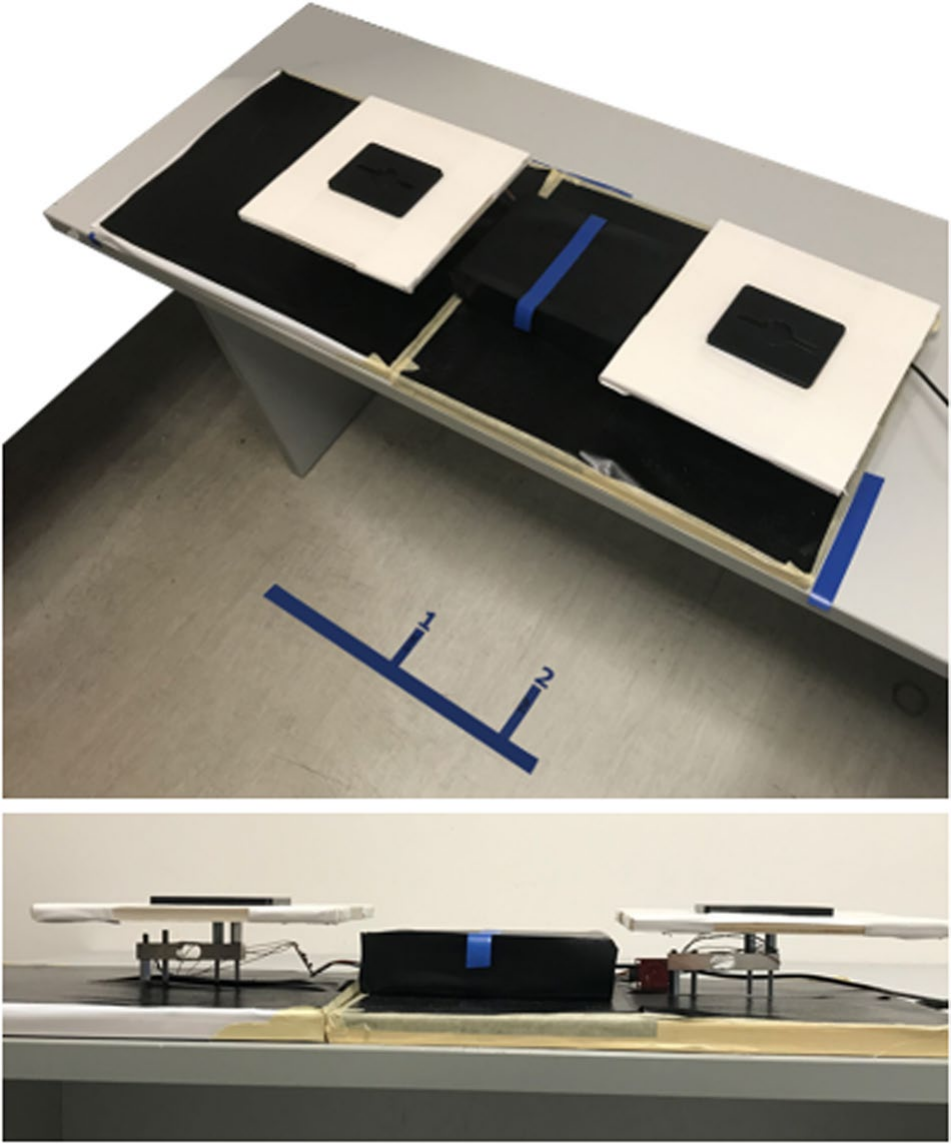
Instrumented platforms. All objects were manipulated over one or two platforms each instrumented by a load cell. The load cells allow tracking of an action’s most salient events like touch, lift-off, replace and release.

All objects were manipulated over one or two instrumented platforms (Fig. 3), each was 9 cm tall from the support desk and with landing areas of 60 cm^2^. When two platforms were used, their landing areas were spaced out by 40 cm. Each platform was instrumented by a strain gauges-based load cell to allow tracking the instant of first contact with the object as well as lift off and replace from/to the platforms. The load cells had 1 kg mass limit and were acquired by HX711 (Avia Semiconductor, China) integrated circuit with an ESP32s2 host MCU (EspressIf, China). Data was streamed at 80 Hz and acquired via wired USB connection.

Moreover, two floor marks were used to guide the participants’ positioning in relation to the instrumented platforms. Specifically, each floor mark would determine a different standing position, thus a different positioning of the proximity sensors’ field of view. Mark #1 was intended to aim the proximity sensors’ field of view to the left platform, thus to have the target object within the fields of view at start position. Mark #2 was intended to align the proximity sensors’ field of view to the centre of the two platforms (indicated with a blue line in Fig. 3), thus to have the target object not necessarily within the fields of view at start position. The marks were found empirically via few representative participants.

#### Data acquisition software

The data from all parts of the equipment were acquired via multiple USB connections to a single USB 3.1 HUB and a portable computer (HP Elitebook 840). The acquisition was coordinated via a custom software written in Matlab (R2022a, Mathworks, USA). The software included a graphic interface for labelling the trials, meant to facilitate the job of the experimenters. Data was acquired in big bulks (i.e., more than one sample per time) so to improve temporal consistency. Time stamps were added automatically to the incoming bulks of data, from which the individual sensor timestamps were then interpolated. The acquired data consisted of proximity, inertial and fingers joint angles measurements from the instrumented glove, and load forces measurements from the instrumented platform.

### Data acquisition protocol

Data was acquired from 29 adult volunteers (5 female) with age 27.4 ± 2.95 y/o and height 171.85 ± 8.26 cm, healthy and without any prior neuromuscular disorder. Two volunteers out of 29 had left dominant hand. Prior to any data acquisition, participants were prepared with the instrumented glove: the glove was worn on the right hand (Fig. 1), carefully positioning the wrist band and wires avoiding any movement restriction. Participants stood in front of a support desk where an instrumented platform was located (Fig. 3). The desk height was 70 cm and it was kept constant across participants with the aim to promote inter-subject data variability in matter of reaching angle and trajectory. Before starting the experiment, a short data recording was acquired for each participant so to provide calibration reference data for all sensors. Specifically for the CyberGlove, participants were asked to go through several hand movements such as rest, hand open, hand close, and flex all fingers sequentially. Then, the experiment was composed of four different phases representing increasing complexity scenarios, namely bench-static, user-static, pick-and-lift and pick-lift-and-move (Fig. 5). The whole experiment took around 30 minutes per participant, of which, about 15 minutes were for setup preparation.

#### Bench-static scenario

**Fig. 4:**
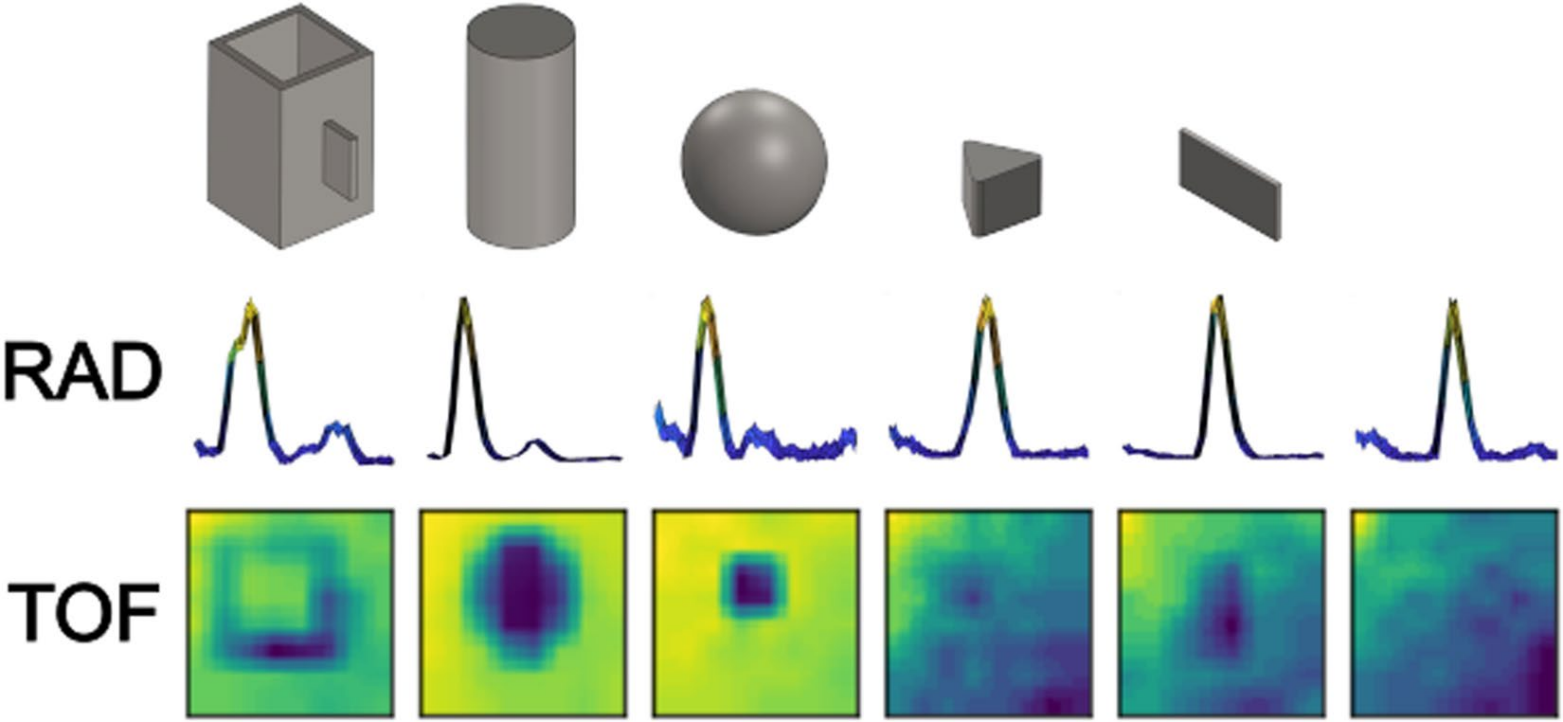
Proximity sensors data. Visualization of the data acquired from the radar and time-of-flight sensors while steadily facing the different target objects. For illustration purposes, we show (1) the normalized amplitude of the radar readings for the 35 range points, thus the distribution of the reflection distances in the available distance range (from left to right, 6 cm up to 41 cm), and (2) the normalized distance readings of the time-of-flight sensor, resized as 35x35pixels and plotted as pseudo-colour images (“viridis” colour mapping).

**Fig. 5:**
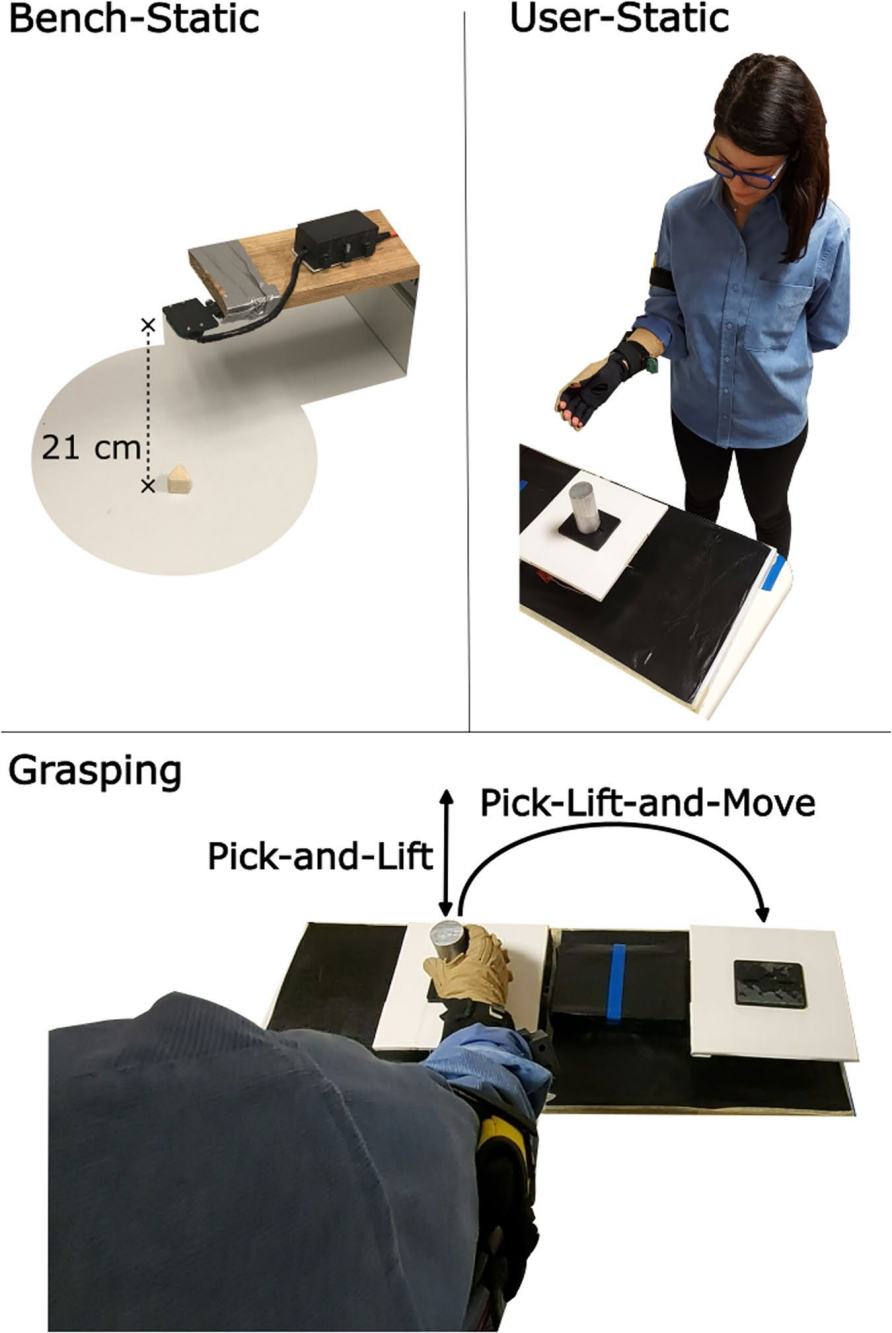
Data acquisition protocol. The data acquisition was composed of four different phases representing increasing complexity scenarios, namely bench-static, user-static, pick-and-lift and pick-lift-and-move. In the static scenarios the sensors were steadily facing the target objects, while in the grasping scenarios the user was interacting with the object. The participant shown in this figure has consented to the use of the image.

With the aim of preliminarily assessing the proximity sensors capabilities for object recognition under ideal conditions, a bench data recording phase was performed, namely bench-static scenario (Fig. 5). Here, sensors were placed at a fixed height pointing downwards on a support desk where all objects were sequentially placed. A horizontal support arm held the sensors at a fixed height of 21 cm, carefully positioned to avoid interfering with proximity data recording. Data from each object was acquired for about 12 seconds split in 6 trials. Following the lead from similar work^26^, objects’ orientation was changed in between trials (see supplementary material for the protocol). Different orientations were deemed necessary in order to capture different cross sections of the same object which may reveal different shapes, thicknesses and densities. An illustrative sample of data acquired from the proximity sensors is shown in Fig. 4.

#### User-static scenario

User-static represents the easiest user scenario, meaning a static situation in which the user is standing in front of the object and the proximity sensors are steadily facing the target located on one instrumented platform (Fig. 5), specifically the left one. Participants were asked to stand in front of the support desk with feet aligned with mark #1 on the floor, keeping the arm in rest position. The rest position was defined for all duration of the experiment as the upper arm adjacent to the body trunk, with elbow joint bent at 90 degrees, with the hand palm pointing left and perpendicular to the floor (see Fig. 5). Participants were asked to keep the hand (i.e., the proximity sensors) steady and facing the target object located around 20 cm away. Around 5 seconds of data were recorded for each of the ten objects.

#### Grasping scenario: Pick-and-Lift

Pick-and-Lift scenario represents a dynamic situation in which the user is picking and lifting the target object, thus the proximity sensors are moving towards the object located on always one instrumented platform (Fig. 5), specifically the left one. Participants were asked to stand in front of the support desk with feet aligned with mark #1 on the floor keeping the arm in rest position, exactly as the static phase. Then, for the recording, participants were asked to repeatedly perform a simple reach-grasp-lift-replace task with the target object. Specifically, the task consisted of (1) reaching the object, (2) grasping the object with its predefined grasp, (3) lifting it in the air of about 10 cm, (4) repositioning it on the starting area and lastly, (5) going back to rest position as previously defined. Task repetitions were organized in 10 blocks of 10 trials each, differentiated by the object shape (i.e., sphere, cylinder, triangular prism, cuboid with thin rectangular prism ‘handle’ and thin rectangular prism) and by its material (i.e., wood or aluminium), as described in Table 1. Participants were invited to perform the grasping actions as naturally as possible, to choose the pace that would be more comfortable to them and lastly to keep the pace between task repetitions as consistent as possible. Moreover, audio cues were provided, as start and stop signals, to support pace consistency. Importantly, each object shape, regardless to its material, was pre-assigned to a specific grip pattern (Table 1) and participants were asked to use that pattern for all grasping actions related to that particular object shape. A test operator was dedicated to constantly verify that the correct grasp was always used, otherwise alerting a second operator in control of the acquisition software to discard the data via a “repeat trial” button.

**Table 1:** Protocol summary.

OBJECT	GRIP PATTERN	MATERIALS	Static	Pick-and-lift	Pick-lift-and-move
Sphere	Spherical	Wood	5 s	10 trials	10 trials
		Aluminium	5 s	10 trials	10 trials
Cylinder	Power	Wood	5 s	10 trials	10 trials
		Aluminium	5 s	10 trials	10 trials
Triangular Prism	Tri-digital	Wood	5 s	10 trials	10 trials
		Aluminium	5 s	10 trials	10 trials
Cuboid with handle	Lateral	Wood	5 s	10 trials	10 trials
		Aluminium	5 s	10 trials	10 trials
Rectangular Prism	Bi-digital	Wood	5 s	10 trials	10 trials
		Aluminium	5 s	10 trials	10 trials

The order of the objects’ shape was always randomized to avoid learning or adaptation effects. Similarly to the SHAP protocol^25^, objects made by wood were performed first, then the same order was repeated with the objects made by metal. Unrecorded training trials were offered before starting with a new object so to allow getting acquainted with new weight and material as well as to facilitate pace selection and consistency.

#### Grasping scenario: Pick-Lift-and-Move

Pick-Lift-and-Move scenario represents a dynamic situation with increased complexity in which the user is picking and transporting the target object (Fig. 5), thus the proximity sensors are moving towards the object from different directions. In fact, here the target object was located alternatively on two different instrumented platforms, thus alternating whether or not the object was within the proximity sensors’ field of view at the task start. Specifically, the left platform was in view at start while the right one was not. The Pick-Lift-and-Move scenario was similar to the Pick-and-Lift, but with the following differences:

- Participants were asked to stand on a different position, with feet aligned with mark #2 on the floor. Here, the arm during rest position was always aligned in between the two instrumented platforms.
- The areas of start (i.e., pick) and landing (i.e., replace) were alternated between task repetitions, meaning the next start area being the previous landing area.

### Ethical approval

All shared data was completely anonymized. All participants signed informed consent for data and media acquisition and public release. The ethical approval was provided by Ethical Committee of the Scuola Superiore Sant’Anna (ref. 12/2022).

## Data Records

HANDdata is freely available on Figshare^18^. The data was recorded and stored via Matlab, thus all files are with *.mat extension. Moreover, to improve accessibility the data was also converted and shared with *.csv extension and organized within an intuitive folder structure.

### Data format

#### *.mat files

Each file is a data structure which includes a list of variables, briefly described in the following:

- *date_time*, of the recording session with format DD-MM-YYYY_hh-mm
- *duration*, of the recording session in seconds
- *id*, of the volunteer participant in range 01–29
- *task*, either “Calibration”, “Static”, “Pick-and-Lift” or “Pick-Lift-and-Move”
- *item*, used for the recording session
- *notes*, added manually by the experimenter, if any
- *trials*, actual raw data

**Fig. 6:**
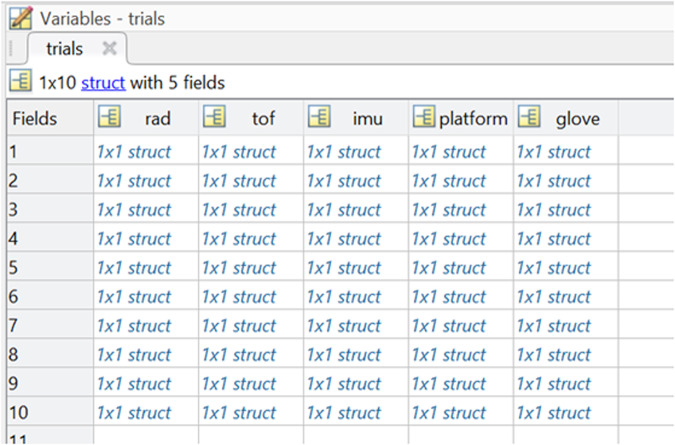
Raw data structure.

**Fig. 7:**
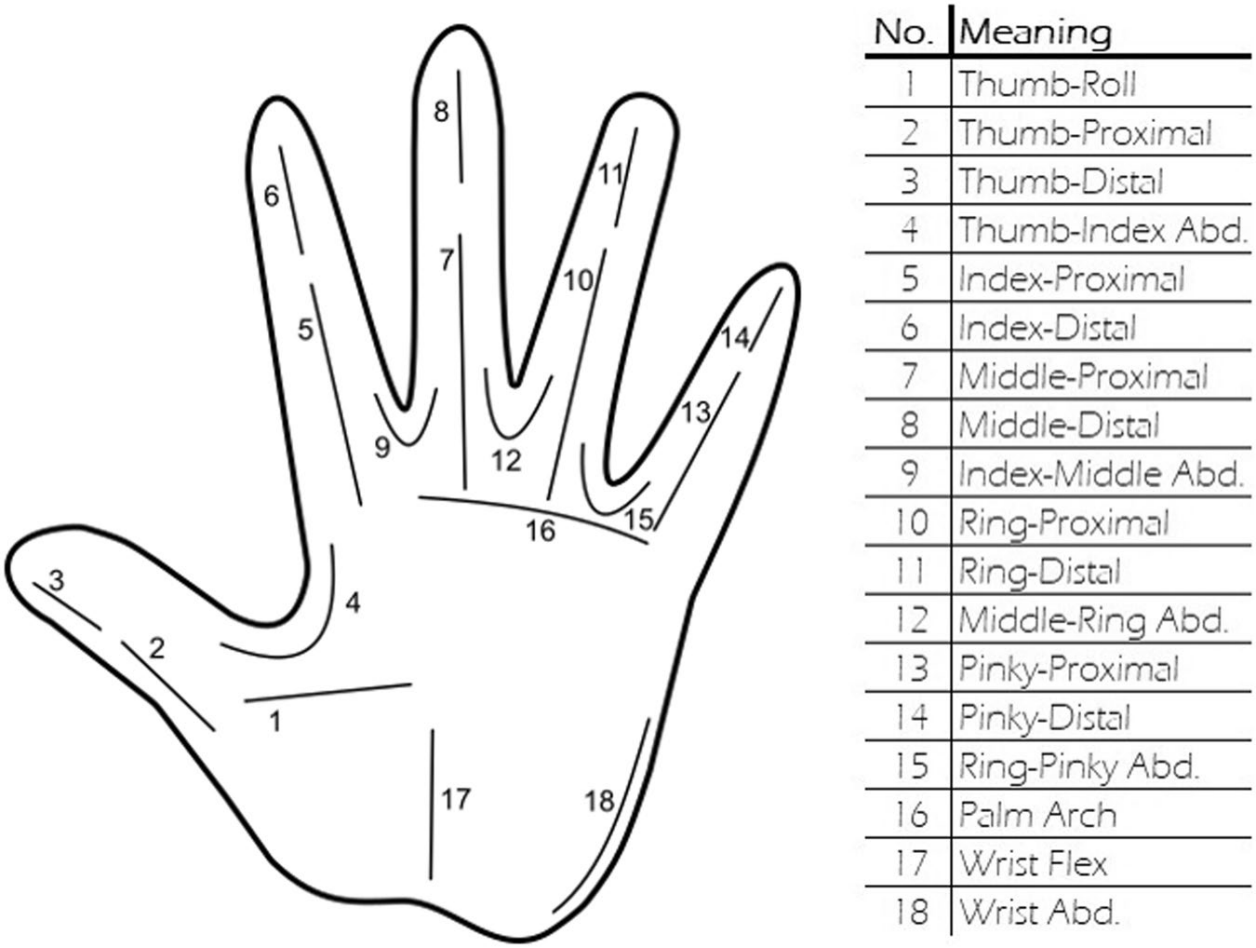
CyberGlove sensors.

Specifically, the raw data is included in the *trials* variable as a matrix where rows represent the trial number and columns represent the sensor (Fig. 6). The sensors’ data is represented as described in the following :

- *rad*, data from radar A121 sized as [num_frames * 40 sweeps_per_frame * 35 range_points_per_sweep]. Specific details about A121 radar’s frames, sweeps and settings can be found in its datasheet and on Acconeer’s online handbook.
- *tof*, data from time-of-flight VL53LCX sized as [num_frames * 64 pixels_per_frame]. Each pixel contains the measured distance from the sensor for that particular area.
- *imu*, data from inertial sensor BMI160 sized as [num_samples * (3 xyz_axes_accelerometer + 3 xyz_axes_gyroscope)].
- *platform*, data from load cells sized as [num_samples * (left platform + right platform)].
- *glove*, data from CyberGlove sized as [num_samples * 18 stretch_sensors]. Specifically, the sensors’ numbering, location and meaning is summarized in Fig. 7.
  Lastly, the raw data from each of these sensors is accompanied by a *time* vector to describe when a particular frame or sample was actually acquired.
  It is worth noting that, for the Pick-Lift-and-Move scenario, there is no explicit mentioning of the starting/ending platforms. However, this information can be easily derived from the number of the trial (odd trials = start on left platform, and even trials = start on right platform).
  Each calibration file is identified by the CAL label in the file name, and it includes 9 trials composed of the following data:
- *Static recording of no object* for around 5 seconds. Participants had the arm extended out, elbow bent at 45° and wrist bent back, aiming at a white wall approximately 1 meter far away.
- *Rest hand* for around 5 seconds. Participants were in rest position, thus with the right upper arm adjacent to the body trunk, with elbow joint bent at 90°, with the hand palm pointing left and perpendicular to the floor.
- *Open hand* and rest.
- *Close hand* and rest.
- *Flex index finger* and rest.
- *Flex middle finger* and rest.
- *Flex ring finger* and rest.
- *Flex pinky finger* and rest.
- *Flex thumb finger* and rest.

The data included in the calibration file can provide sensors’ reference values for each subject, such as baselines and variation ranges. We summarised this information in Table 2.

**Table 2:** Calibration file summary.

What?	Where?	Who?	How?	Why?
no object - no background	Trial 1	Radar and TOF	Pointing the proximity sensors at no target towards a white wall located beyond the sensors maximum range	No object class data, background removal techniques
no object – with background	Trial 2	Radar and TOF	Pointing the proximity sensors at the empty left platform	No object class data, background baseline
No movement	Trial 2	IMU	Keeping the arm (and IMU) steady in default rest/start position	IMU no movement baseline
Resting hand baseline	Trial 2	CyberGlove	Keeping the arm and hand (and stretch sensors) steady in default rest/start position	Rest hand baseline, compensate to different glove fit for each subject
Moving hand ranges	Trial 3 to 9	Cyberglove	Performing a sequence of hand movements	Stretch sensors variations for all channels

#### *.csv files

The description related to files in *.mat format is almost entirely applicable to the *.csv format, with the following differences:

- the data is organized as different files within an intuitive folder structure.
- Each sensor for each trial has a dedicated file which always includes the *time* vector as the first column.
- *rad*, data from radar A121 sized as [num_frames * 1400 sweeps_per_frame * range_points_per_sweep], organized as (fr1,sw1,rp1) (fr1,sw2,rp1) (fr1,sw3,rp1).

### Folders structure

#### *.mat files

Recorded data is structured in 30 different folders, one dedicated to the bench-static data and the rest dedicated to the 29 volunteer participants. The bench-static folder includes 10 files, one for each object. The participants’ folders include 31 files, one for the calibration session and the rest for each task and object (i.e., 3 tasks * 10 objects).

Each file in these folders was named as <*id_task_item-material_date-time*> where each piece of information is described as above. For instance, the file “02_PLM_box-wood_3-11-2022_15-26.mat” includes the recording “Pick-Lift-and-Move” with the wooden box of the volunteer participant number 02.

#### *.csv files

The folders structure for the *.csv files follows similar hierarchical logic applied to the *.mat data structures. Recorded data is structured in 30 different main folders, one dedicated to the bench-static data and the rest dedicated to the 29 volunteer participants. Inside each main folder, there are subfolders dedicated to each scenario (or task). Then, going one step further, there are subfolders dedicated to each object within that scenario. All informative variables, such as *date_time*, *duration*, *id*, *task, item and notes* are included here in a dedicated *.txt file. Lastly, each of these folders include separate *.csv files for each sensor.

## Technical Validation

In the following section we present several analyses aimed to validate the acquired data. All data were analysed using the “data_validation” script and available built-in functions of MATLAB (R2022b, MathWorks).

**Fig. 8:**
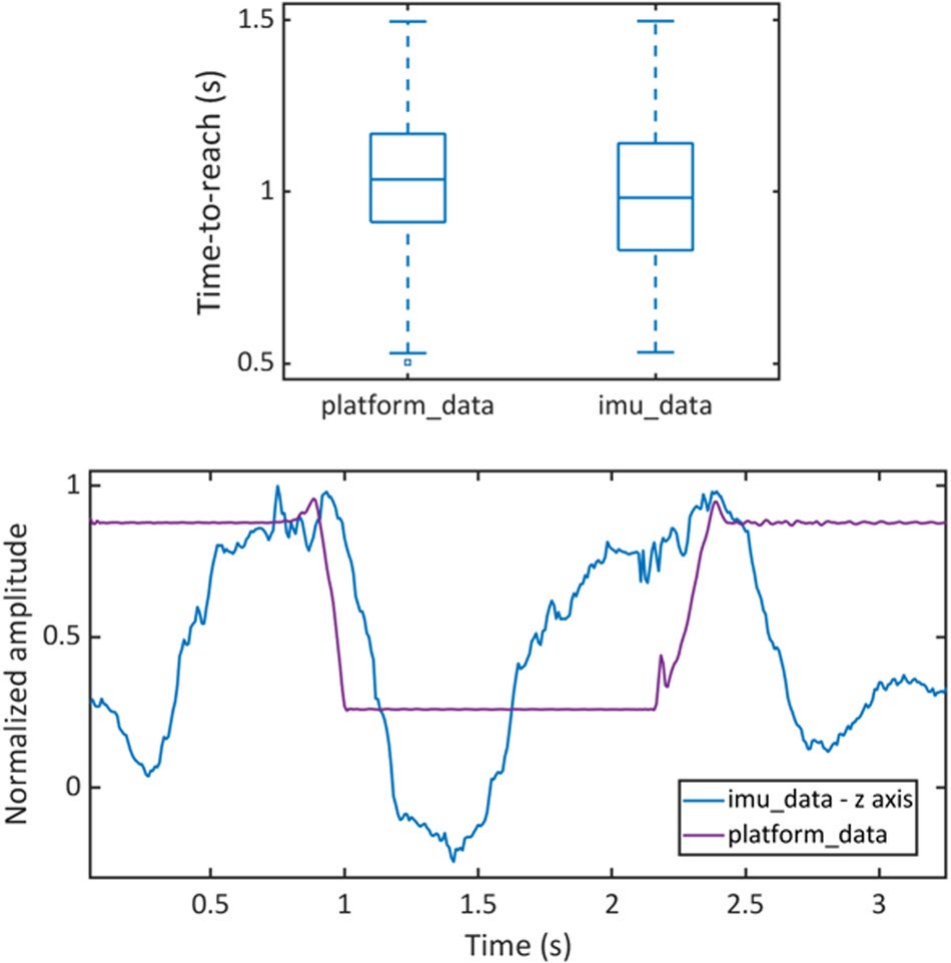
Validation of grasping trials, platform and inertial sensors data. Time-to-reach the target object computed for all pick-and-lift trials from both the platform and the inertial sensor data (top). A representative trial of a pick-and-lift action with the metallic cylinder as target (bottom).

Firstly, in order to validate the dynamics of the pick-and-lift trials, we computed the time-to-reach defined as the time between the start of the trial and the reach of the target object (Fig. 8, top). The time-to-reach was computed from both the platform and the inertial sensor data. For the platform data, the instant of touch was found as the maxima in the first half of the trial. For the inertial sensor data, the instant of touch was approximated as the first maxima in the z-axis, thus the instant of zero in the acceleration towards the target object. From the platform data, participants reached the target in 1.04:0.26 seconds (MEDIAN:IQR). For the sake of completeness, we include a representative trial of a pick-and-lift action with the metallic cylinder as target (Fig. 8, bottom).

**Fig. 9:**
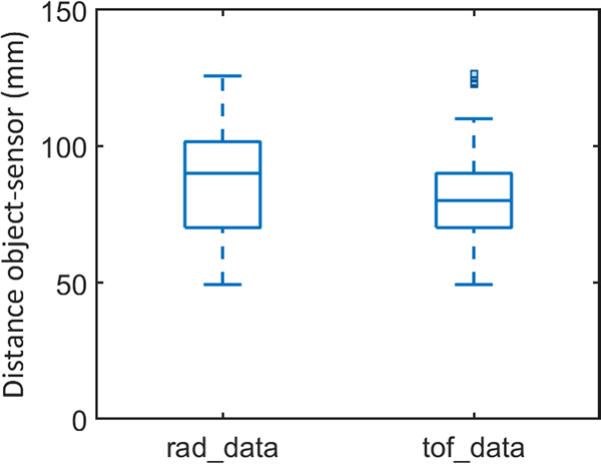
Validation of static trials, radar and time-of-flight sensors data. Comparison of minimum measured distances for static trials.

Secondly, in order to validate the static trials and the data from the radar and of the time-of-flight sensors, we calculated their minimum measured distances for the static-user trials (Fig. 9). The aim was to prove that the target objects were indeed within each sensor range of view, approximately around 10–15 cm away from the sensors. Values lower than these and general variability can be attributed to sensors’ readings on the participants’ forearm and on the platform where the target objects were located.

**Fig. 10:**
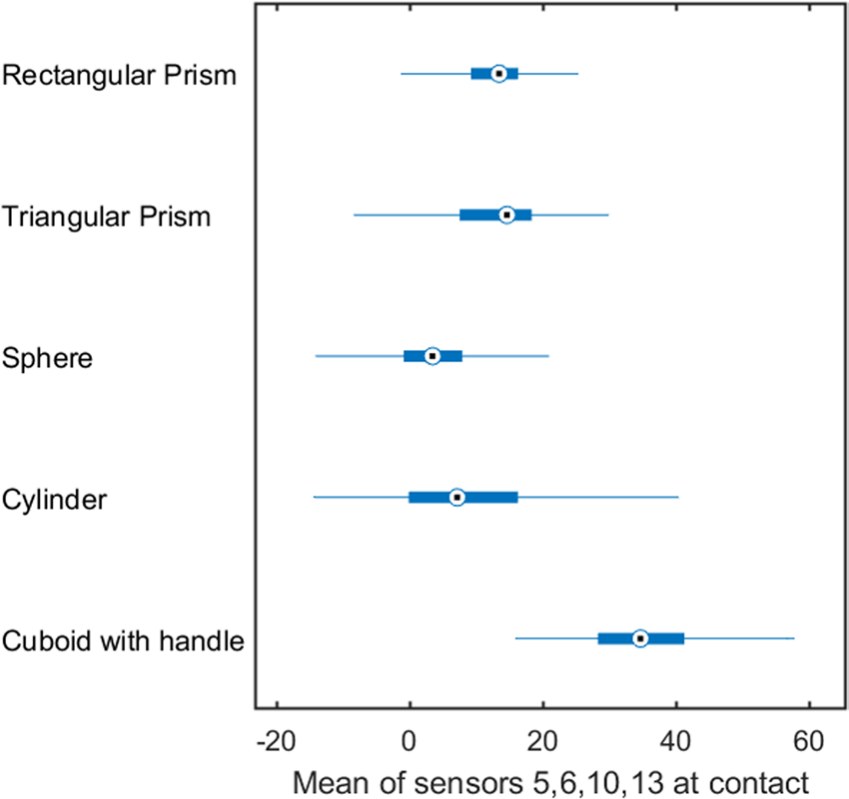
Validation of CyberGlove stretch sensors data. Mean of the most representative sensors values (i.e., 5, 6, 10, 13) upon contact with each target object during grasping trials.

Thirdly, in order to validate the CyberGlove data, we computed the mean of some of the most representative sensors values, as suggested in previous literature (i.e., sensors 5, 6, 10, 13 as in^27^), at contact with the target object in grasping trials (Fig. 10). The aim here was to visually demonstrate some basic patterns in the final position of the index, ring and pinky fingers when grasping objects with different size and requiring different hand posture. Specifically, higher readings would correspond to stronger stretches of the sensors in respect to the rest position (i.e., larger flexion span of the index, ring and pinky fingers). Indeed, the tri-digit and pinch grips required by the prisms produced similar averaged flexion span. Similarly, we can see close averaged flexion span for the spherical and power grips. Instead, the lateral grip for the cuboid with handle required a complete flexion of the index, ring and pinky fingers thus producing higher averaged readings.

Unfortunately, during technical validation of the data we realized that one of the 18 stretch sensors of the CyberGlove was not functional. This sensor relates to the thumb roll movement, for which, sadly, we cannot include any data. Nevertheless, we believe that the thumb position during the recorded actions can be decently represented by its 3 remaining sensors.

Lastly, it is important to note that our setup did not have hardware synchronization lines. Consequently, the temporal synchronization of our data relied on timestamps generated for each incoming bulk of data on the USB. However, this approach can bring potential disadvantages in matter of high latency and poor determinism. Thus, we analysed the temporal consistency of the acquired bulks of data. We found the interquartile range of the time distance between bulks to be 10.43 ms for the MCU-based custom PCB providing IMU, radar and TOF data, 8.36 ms for the MCU-based custom PCB providing the platforms load cells data, and 5.87 ms for the CyberGlove II. Therefore, we argue that the data streaming had temporal consistency and overall desync error suitable for a human-motion dataset.

## Supplementary information

Bench-static Protocol

## Data Availability

Together with the dataset, we also provide the “data_validation” Matlab script used for technical validation of the data. This script is accompanied by helpful comments and can be used as example for how to access and handle the data (e.g., how to access the data structure, how to loop through participants, scenarios and trials, how to access certain trial info, how to find the instants of touch via IMU or via the platform).
