# Supplementary material for: HANDdata – first-person dataset including proximity and kinematics measurements from reach-to-grasp actions
Source: Sci Data. 2023 Jun 24;10:405. doi: 10.1038/s41597-023-02313-w (PMC10290694; doi:10.1038/s41597-023-02313-w)

Bench-static scenario protocol for objects' orientation

The different orientations were set up as to reveal all unique features and/or dimensions of each object. Uniformly shaped objects (such as the sphere) will not have unique features and/or dimension in different orientations but for data size consistency the object was still recorded in different orientations. The density of the (more) uniform objects may not be uniform so that the different orientations will not provide superfluous information.

All the figures in this document are shown from the sensor's point of view (from above). The order in which they're discussed and numbered in the images is the same as in the trials of the data provided by the article.

Triangular prism

In the first trial, the prism was stood up on the triangular face with the apex pointing up. The second trial was done similarly with the apex pointing down. These simple rotations may seem redundant for deep learning applications like convolutional neural networks since they are impervious to image translations. In the case of a radar, such a simple rotation can change the returning signal so it is necessary to include these in training with radar data. The third trial is done by laying the triangular prism on the rectangular face with the apex (elongated in orange) pointing towards the sensors. Trial 4 is a simple 90° rotation of trial 3. Trials 5 and 6 are very similar to 3 and 4 except that they are done by balancing the triangular prism on the orange edge. The edges are flattened, unlike the sharp peaks shown in orientations 1 and 2, thus easing the object balancing.

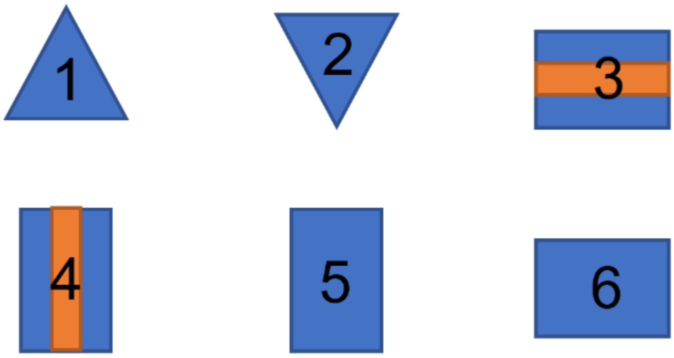

Thin rectangular prism

The first two trials were done by laying the thin rectangular prism flat on the desk and doing a simple 90° rotation. Trials 3 and 4 were done by balancing the rectangular prism on the long edge and then rotating it by 90°. Trials 5 and 6 are similar but now the rectangular prism is balanced on the shorter edge which means it stands taller and is thus closer to the sensors than in trials 3 and 4.

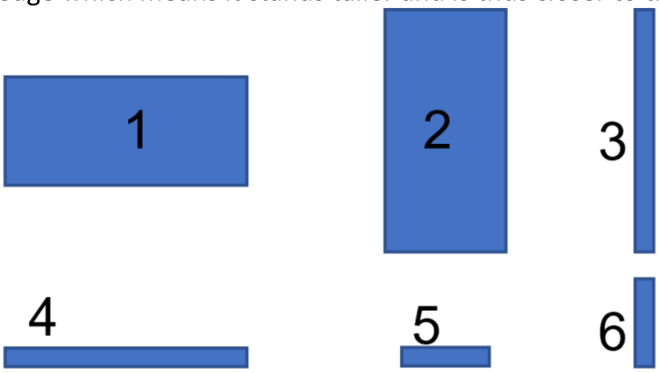

Cuboid with a thin rectangular prism 'handle'

For simplicity, the cuboid will be referred to as a box and the orange rectangle in the image is the handle. In the first two trials, the box was laying on one of its rectangular faces with the handle pointing straight toward the sensors (trial 2 was done by rotating the box by 90°). Trials 3 and 4 are similar except that the box is now laying on a rectangular face so that the handle is now in view when viewed from above. Trial 4 is a simple 90° rotation from trial 3. Trials 5 and 6 were done by standing the box up on its square face instead of its rectangular face so that the thin face of the handle was visible to the radars (as they are shown in figure 2). Doing so also decreases the distance between the box and the sensors. It is important to note that the wooden box object had a panel at the square face while the metal box object was completely hollow throughout (like a tunnel). To minimize the variability that this causes the wooden box was placed with the panel on the desk in trials 5 and 6.

This object could have used more trials like two additional 90° rotations for orientations 3-6 but this would create a large discrepancy in the sizes of the data between the rest of the objects. Furthermore balancing the box on the handle was not possible without the use of additional tools so they were not considered.

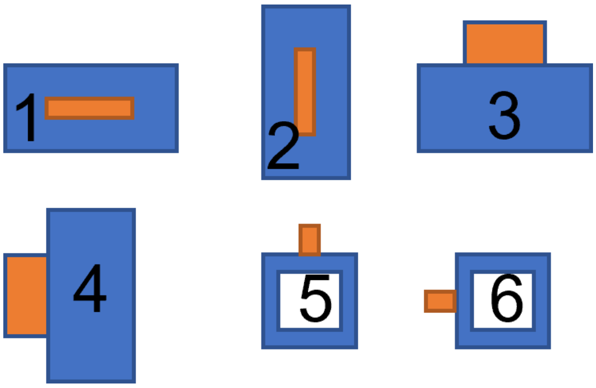

Cylinder

The N and S on the circular faces are fixed throughout the figure so that the face marked with an N will never change. If the N or S are not centered it means the face marked with the N or S is at the location marked in the image but not visible to the sensors. In the first trial, the cylinder stands on the circular S face showing the N face to sensors (similar to how it stands in figure 2). The second trial is simply a repetition of the first one flipped upside down so that the S face is now shown to the sensors.
In trial 3 the cylinder is carefully balanced by laying it flat on the desk with the S and N faces shown in the image. From trial 3 onwards the cylinder is rotated clockwise by 90° each trial.

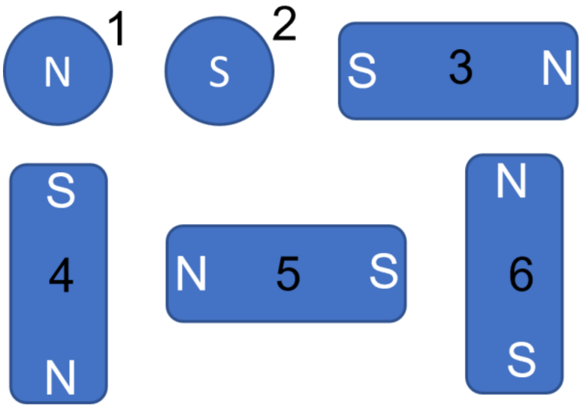

Sphere

Equivalent to the cylinder the sphere was marked with a N and S to indicate how the rotations took place. Just like with the cylinder if the S or N are not centered in the middle of the object they are not visible to the sensors but are there only to illustrate which direction they are pointing in. Furthermore, all the rotations were carried out in the direction indicated by the arrows in the image.
For the actual experiment, the sphere was marked with a small dot to indicate where the N was so that the rotations could be carried out. The starting position was arbitrarily set and then marked with the dot. Then in trial 2, the sphere was rotated by 90° so that neither the N nor the S was technically visible to the sensors. In trial 3, a subsequent 90° rotation reveals the S to the sensors. For trial 4 another 90° rotation was carried out. From trial 4 onwards the sphere was rotated by 120° but from a new direction as shown by the arrows.

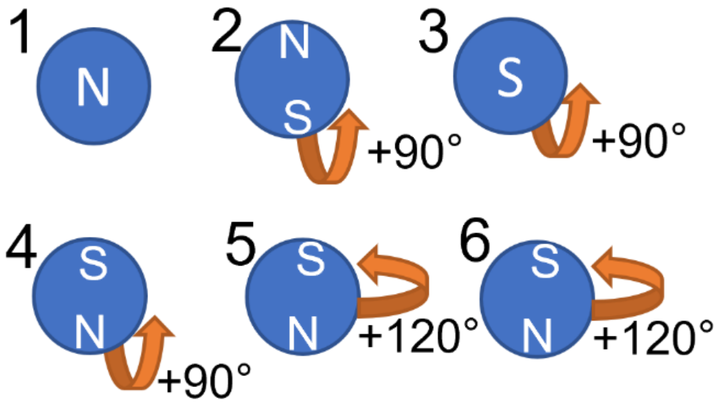

Supplement: Supplementary file 1 — Bench-static Protocol [file 41597_2023_2313_MOESM1_ESM.pdf]
